# Resurgence of Pertussis in the Autonomous Province of Vojvodina, Serbia: Shifting Seasonality, Age Patterns, and the Need for Booster Immunization

**DOI:** 10.3390/vaccines13080814

**Published:** 2025-07-31

**Authors:** Mioljub Ristić, Vladimir Vuković, Smiljana Rajčević, Snežana Medić, Marko Koprivica, Vladimir Petrović

**Affiliations:** 1 Institute of Public Health of Vojvodina, 21000 Novi Sad, Serbia; smiljana.rajcevic@mf.uns.ac.rs (S.R.); snezana.medic@mf.uns.ac.rs (S.M.); marko.koprivica@izjzv.org.rs (M.K.); vladimir.petrovic@mf.uns.ac.rs (V.P.); 2 Department of Epidemiology, Faculty of Medicine, University of Novi Sad, 21000 Novi Sad, Serbia

**Keywords:** pertussis, surveillance, seasonality, age-distribution, immunization, AP Vojvodina, Serbia

## Abstract

Background: Despite decades of high childhood vaccination coverage, pertussis has re-emerged in the Autonomous Province of Vojvodina (AP Vojvodina), Serbia. We aimed to describe the temporal, seasonal, and age-specific patterns of pertussis in AP Vojvodina and to analyze trends by vaccination status in order to highlight changes in epidemiology and potential gaps in vaccine-induced protection. Methods: We retrospectively analyzed 2796 pertussis cases reported between January 1997 and December 2024, examining temporal, seasonal, and age-specific trends, stratifying by vaccination status across four consecutive periods (1997–2003, 2004–2010, 2011–2017, and 2018–2024). Results: Throughout the 28-year period, after low and sporadic cases in the pre-2012 period, a dramatic rise was observed in 2014, 2017, and 2018, culminating in the highest annual number of reported cases in 2024 (1011 cases). Throughout this period, primary vaccination coverage with the DTwP/DTaP three-dose series ranged between 91% and 98%, while first booster coverage gradually declined from 98% in the early 2000s to 83% in 2024. Regarding seasonality, a sharp increase in cases began in 2012, peaking in November 2023 (>350 cases) and early 2024 (312 in January, 268 in February), with a seasonal shift from summer peaks in the 2011–2017 period to higher incidence rates during colder months more recently. Adolescents aged 10–14 years had the highest cumulative incidence (1149.4/100,000), followed by infants under 12 months (978.5/100,000), despite the latter representing fewer absolute cases. The proportion of pertussis in fully vaccinated individuals rose from 6.3% (1997–2003) to 49.7% (2018–2024). Conclusions: These findings suggest that booster immunization in adolescence and routine maternal vaccination during pregnancy could reduce transmission, particularly to infants. Enhanced surveillance and updated immunization policies are critical to mitigating future pertussis outbreaks.

## 1. Introduction

Pertussis, or whooping cough, is a highly contagious respiratory disease caused by Bordetella pertussis (*B. pertussis*), characterized by paroxysmal coughing and a prolonged clinical course. Despite widespread immunization, pertussis remains a public health concern globally, with cyclical outbreaks and a notable epidemiological shift toward older children, adolescents, and adults [1,2].

Although infant morbidity and mortality have traditionally driven pertussis prevention strategies, recent decades have seen increasing recognition of disease burden in older age groups. Waning immunity following childhood vaccination, incomplete booster coverage, and atypical clinical presentations in adults contribute to ongoing transmission and diagnostic challenges [3,4,5,6].

In the last two decades, several countries have reported the resurgence of pertussis, often associated with improved diagnostic techniques, enhanced surveillance, and changes in vaccine formulations [1,7,8]. The COVID-19 pandemic, with its impact on healthcare-seeking behavior and public health interventions, has further complicated pertussis surveillance and control efforts [9].

In Serbia, pertussis is a mandatory notifiable disease, and mandatory routine immunization has been implemented for decades [10,11]. However, limited studies have explored long-term epidemiological trends, seasonality, or age-specific patterns of pertussis in the region. The Autonomous Province of Vojvodina (AP Vojvodina), representing about a quarter of Serbia’s population, maintains a comprehensive infectious disease surveillance system, making it an ideal setting for such analyses.

This study aims to describe the temporal, seasonal, and age-specific distribution of pertussis cases in AP Vojvodina from 1997 to 2024. Additionally, we analyze trends by vaccination status across four time periods, providing insight into the shifting epidemiology and potential gaps in vaccine-induced protection.

## 2. Materials and Methods

### 2.1. Study Design and Setting

We conducted a retrospective observational study analyzing pertussis cases reported in AP Vojvodina, Serbia, over the 28-year period, from January 1997 to December 2024. AP Vojvodina, an autonomous province in northern Serbia, has a population of approximately 1.9 million. As previously described [12], it maintains an integrated infectious disease reporting system coordinated by the Institute of Public Health of Vojvodina (IPHV).

### 2.2. Case Definition and Surveillance

Pertussis has been a notifiable disease in Serbia since 1948. As detailed in our earlier work [12], prior to 2012, pertussis cases were reported by healthcare providers based primarily on clinical criteria, and the majority of cases were hospitalized patients. In 2012, an improved pertussis surveillance system was implemented in AP Vojvodina in accordance with the Global Pertussis Initiative (GPI) recommendations, incorporating standardized clinical case definitions and laboratory confirmation of cases among both outpatients and hospitalized patients [13,14].

### 2.3. National Immunization Program in Serbia

Mandatory vaccination against pertussis in Serbia was introduced in 1960. Details on the primary immunization series and booster doses are presented in Table S1 [10,11,15,16]. In addition to these vaccines, boosters against pertussis for adolescents, adults, pregnant women, and healthcare workers (HCWs) were included in the Serbian National Immunization Program; however, these vaccines are currently not available [15,16].

### 2.4. Data Collection

Data on all notified pertussis cases, as well as immunization coverage (primary immunization during the first year of life and the first booster dose during the second year), were extracted from the centralized IPHV surveillance database for the study period. For each pertussis case, we collected the following variables: month of notification, age at diagnosis, and vaccination status. Monthly case counts were analyzed in total and stratified into six age groups: ≤12 months, 1–4 years, 5–9 years, 10–14 years, 15–19 years, and ≥20 years.

### 2.5. Statistical Analysis

Descriptive statistics were used to assess temporal trends, cumulative incidence, and seasonal patterns. Annual pertussis incidence rates were calculated using the number of registered cases (including both outpatients and inpatients) as the numerator and the annual population estimates for AP Vojvodina as the denominator [17], multiplied by 100,000. Average incidence rates for total pertussis cases (outpatients and inpatients) were calculated by dividing the cumulative number of cases (crude incidence) by 28, representing the number of years in the study period.

The study period was divided into four intervals for trend analysis: 1997–2003, 2004–2010, 2011–2017, and 2018–2024. Monthly and seasonal variations were assessed for the entire study period and stratified by age group. Vaccination status against pertussis was analyzed within these four intervals and classified as unvaccinated, incompletely vaccinated (received some doses of pertussis vaccine), fully vaccinated (received all age-appropriate doses of pertussis vaccine), or unknown (no medical documentation of pertussis vaccination).

All annual data were collected through collaboration between the IPHV and the six district Institutes of Public Health located in Subotica, Sombor, Pančevo, Sremska Mitrovica, Kikinda, and Zrenjanin.

## 3. Results

### 3.1. Monthly Pertussis Notifications and Immunization Coverage, 1997–2024

**Figure 1:**
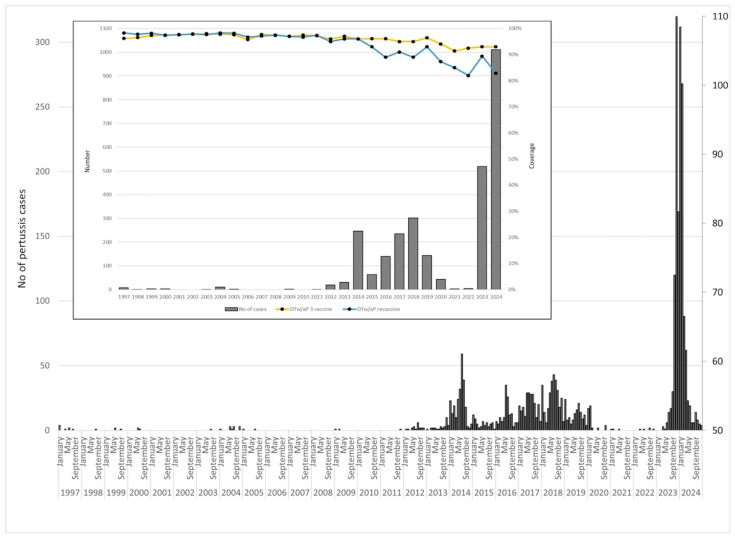
Number of pertussis cases, temporal distribution, and coverage of immunization against pertussis in AP Vojvodina, 1997–2024.

Between 1997 and 2024, 2796 pertussis cases were reported in AP Vojvodina. Cases were rarely registered before 2012 but increased sharply afterward, peaking in 2014, 2017, and 2018. The number of pertussis cases declined during the COVID-19 pandemic (2020–2022) but surged again post-pandemic, reaching 1011 cases in 2024. In addition, between 2014 and 2019, pertussis cases steadily increased, peaking in the summers of 2016–2018. Cases declined during 2020–2021 with the COVID-19 pandemic but resurged sharply from late 2022, reaching over 350 cases in November 2023. This upward trend continued into early 2024, with high case numbers reported in January and February. Throughout the entire period, coverage with the primary three-dose series vaccine (DTwP/DTaP) against pertussis ranged from 91% to 98%. The highest coverage (98%) was recorded in multiple years between 2001 and 2005, while the lowest (91%) was observed in 2021. Revaccination coverage (first booster dose) was similarly high during the early years, with values between 97% and 98% in the period 1997–2011. However, a gradual decline in revaccination coverage was observed thereafter, decreasing from 96% in 2014 to 82% in 2022 and 83% in 2024 (Figure 1).

An analysis of the cumulative monthly distribution of pertussis cases reveals a distinct seasonal pattern, with the highest number of cases occurring during the colder months. Out of the total registered pertussis cases (2796), the highest number of cases was recorded in January, with 458 (16.4%) reported cases, closely followed by November (448 cases, 16.0%) and February (353 cases, 12.6%) (Figure S1).

### 3.2. Age- and Month-Specific Distribution of Pertussis, 1997–2024

**Figure 2:**
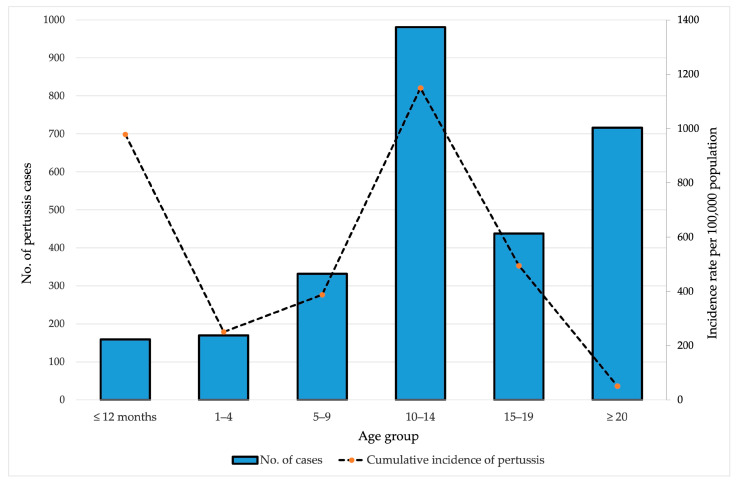
Age-specific distribution of pertussis in AP Vojvodina, 1997–2024.

The age-specific distribution of pertussis cases reported between 1997 and 2024 highlights a pronounced disease burden among adolescents and infants. The highest cumulative incidence was observed in the 10–14-year age group, with 1149.4 cases per 100,000 population, accounting for 981 reported cases. Infants under 12 months of age had the second-highest cumulative incidence, at 978.5 per 100,000, despite representing a smaller absolute number of cases (*n* = 159). The cumulative incidences among children aged 1–4 years, 5–9 years, and adolescents aged 15–19 years were lower (250.9, 387.7, and 494.8 per 100,000 population, respectively). Conversely, the adult population (≥20 years) exhibited the highest number of reported cases overall (*n* = 716) but the lowest cumulative incidence (51.3/100,000) (Figure 2).

Analysis of the monthly distribution of pertussis cases in AP Vojvodina reveals marked seasonal and age-specific trends. The highest overall burden was recorded in the colder months, particularly from November to February, with notable differences across age groups.

**Figure 3:**
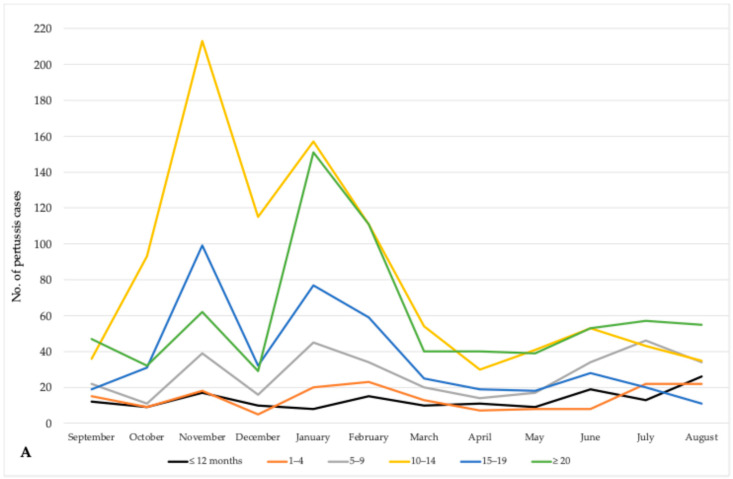

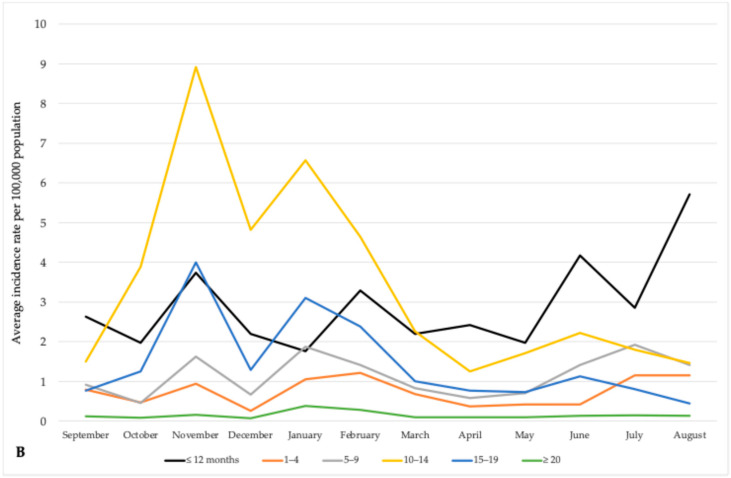
Number of registered pertussis cases by age groups and months (**A**) and average age-specific incidence rates of pertussis by months (**B**) in AP Vojvodina, 1997–2024.

Adolescents aged 10–14 years had a peak average incidence in November (213 cases; 8.9/100,000), with a high number of cases in January and December also. Infants under 12 months showed the highest average incidence in August, June, and November, indicating year-round vulnerability, likely due to household exposure. Adolescents 15–19 years followed a similar pattern with peaks in November, January, and February. Children 5–9 years had relatively stable incidence with slight increases in January, July, and November. The 1–4 years group peaked in February, July, and August but had lower incidence overall. Adults ≥ 20 years had steady cases year-round, peaking in January, though with low incidence rates (Figure 3A,B).

In the Supplementary Materials, we present the distribution of pertussis cases by month and across six age groups (≤12 months, 1–4 years, 5–9 years, 10–14 years, 15–19 years, and ≥20 years) in AP Vojvodina during the period 1997–2024 (Figure S2a–f). A significant increase in the number of pertussis cases has been evident across all age groups since 2012, regardless of seasonal variation.

### 3.3. Distribution of Pertussis Cases by Month, Age and Vaccination Status Against Pertussis Across Four Distinct Periods

**Figure 4:**
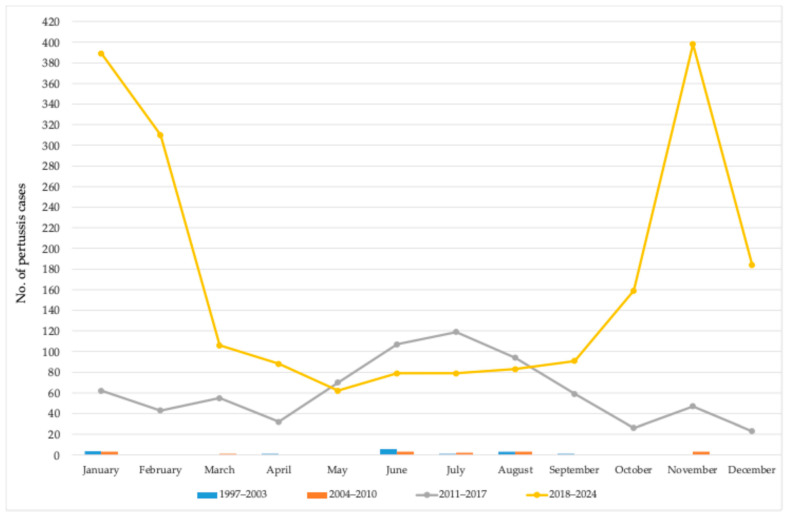
Monthly distribution of pertussis cases in AP Vojvodina from 1997 to 2024 across four distinct periods.

Furthermore, the monthly distribution of pertussis cases in AP Vojvodina was analyzed separately across four distinct periods: 1997–2003, 2004–2010, 2011–2017, and 2018–2024. In the first two periods (1997–2003 and 2004–2010), the number of reported cases remained low throughout the year, with no clear seasonal pattern. In contrast, the third period (2011–2017) marked a substantial increase in the number of cases, with a noticeable rise during the summer months, peaking in June (107 cases), July (119 cases), and August (94 cases). The upward trend continued in the most recent period (2018–2024), during which case numbers increased significantly in almost all months. Notably, the highest peaks were observed in January (389 cases) and November (398 cases) (Figure 4).

In addition, pertussis cases in AP Vojvodina were analyzed across six age groups (≤12 months, 1–4, 5–9, 10–14, 15–19, and ≥20 years) and four distinct time periods: 1997–2003, 2004–2010, 2011–2017, and 2018–2024. A clear shift in the age distribution of cases was observed over time.

**Figure 5:**
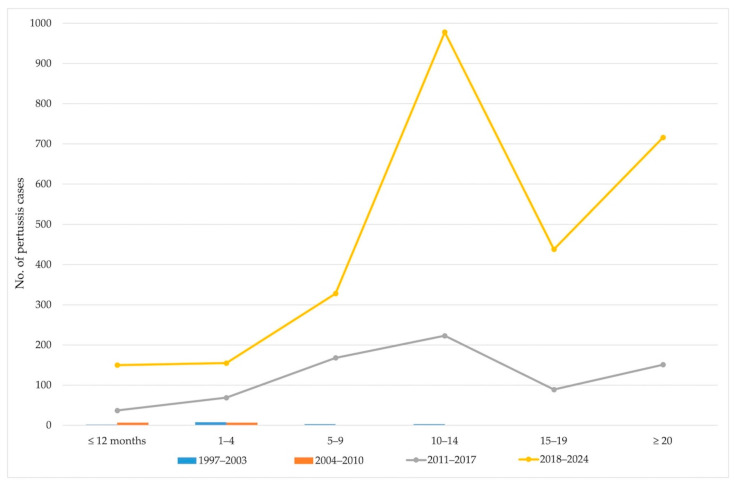
Age distribution of pertussis cases in AP Vojvodina from 1997 to 2024 across four distinct periods.

During the first two periods (1997–2003 and 2004–2010), the total number of cases remained low across all age groups. The majority of cases (77.4%) occurred among children aged ≤4 years, and no cases were reported among individuals aged ≥15 years. In the third period (2011–2017), there was a marked increase in cases across all age groups, with the highest number reported among children aged 10–14 years (223 cases), followed by those aged 5–9 (168 cases), and adults ≥ 20 years (151 cases). This trend intensified in the most recent period (2018–2024), during which the number of pertussis cases rose sharply in almost all age groups. The most affected group was, again, 10–14 years (755 cases), followed by adults ≥ 20 years (565 cases), and adolescents aged 15–19 years (349 cases) (Figure 5).

**Figure 6:**
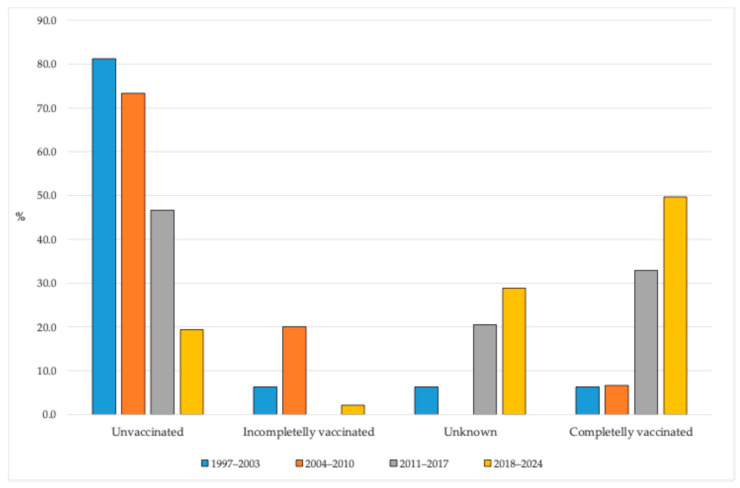
Vaccination status against pertussis in AP Vojvodina from 1997 to 2024 across four distinct periods.

Analysis of the vaccination status of reported pertussis cases in AP Vojvodina, stratified by four time periods, revealed notable temporal shifts in the proportion of cases by immunization status. In the earliest period (1997–2003), the majority of cases (81.3%) occurred among unvaccinated individuals, while only a small proportion were completely vaccinated (6.3%). A similar pattern was observed in the 2004–2010 period, with 73.3% of cases unvaccinated and 6.7% fully vaccinated; however, the share of incompletely vaccinated individuals increased to 20.0%. Starting from the period 2011–2017, a substantial change was observed: The proportion of unvaccinated cases decreased to 46.7%, while the percentage of completely vaccinated individuals among cases rose to 32.8%. Additionally, 20.5% of cases had unknown vaccination status. In the most recent period (2018–2024), the majority of reported pertussis cases occurred in individuals who were either completely vaccinated (49.7%) or had unknown vaccination status (28.8%), while unvaccinated individuals represented less than one-fifth of all cases (19.4%) (Figure 6).

## 4. Discussion

Our findings demonstrate notable temporal, seasonal, and demographic changes in the epidemiology of pertussis in AP Vojvodina over the 28-year study period. In the first two distinct periods, pertussis was primarily characterized by sporadic notifications, with no reported cases in six separate years (2001, 2002, 2006–2008, and 2010). However, pertussis re-emerged with increasing intensity beginning in 2012, coinciding with the implementation of enhanced surveillance measures, including the introduction of GPI case detection and the availability of laboratory confirmation of pertussis, as previously described in detail [14]. This resurgence was particularly pronounced among adolescents and adults [12,14].

A distinct upward trend in pertussis incidence has been observed since 2014, culminating in a dramatic outbreak during 2023–2024. As previously described in detail [12], this dramatic resurgence can be attributed to several factors: a decline in pertussis immunization coverage during the COVID-19 pandemic period; relaxation or absence of measures aimed at controlling the transmission of respiratory infections after decreased intensity of the SARS-CoV-2 virus in population; accumulation of a susceptible population that did not come into contact with *B. pertussis* during the first three years (2020–2022) of the pandemic; waning immunity among previously vaccinated individuals; and increased public awareness and greater availability of pertussis testing after the COVID-19 pandemic.

Throughout the study period, the sharp rise in cases in November 2023 coincided with other respiratory infections [18]. Seasonal analysis (1997–2024) showed a winter predominance with peaks in January, November, and February. However, during 2011–2017, increased transmission occurred in summer (June–August), shifting to late autumn and winter in 2018–2024. Similar summer peaks were reported in Iran (2011–2013) among infants < 2 months [19], Italy (2000–2009) [20], and the USA (1990–2003) [21], despite high vaccination coverage [19,20]. The reasons for this seasonal occurrence of pertussis are not yet fully understood. It is well known that the reopening of schools and the resultant crowding of susceptible students is one of the main factors contributing to the seasonal spread of pertussis. However, the above-mentioned studies [19,20,21], as well as the findings from our study for the period 2011–2017, showed that pertussis was more common in the spring-to-summer months compared to other times of the year. Notably, although only 31 pertussis cases were registered during the 1997–2010 period in AP Vojvodina, 58.1% of these cases occurred between June and August. It appears that these patterns coincide with high childhood vaccination coverage against pertussis, that is, high vaccination coverage may increase the interepidemic interval by reducing the effective reproduction number [19,22]. Specifically, our results showed that the average coverages for primary immunization and the first booster dose against pertussis between 1997 and 2017 were 97% and 96%, respectively. Moreover, during the endemic period, pertussis is likely to be more readily identified in the summer months, as the hallmark cough is less prone to being misattributed to other respiratory infections, which typically decline during this time of year [14].

On the other hand, there is also evidence that pertussis has been more frequently detected during the autumn and winter months, especially after the COVID-19 pandemic. These findings have been reported in several European countries, China, and the USA, highlighting that many immunization activities were missed due to logistical challenges caused by the COVID-19 pandemic [23,24,25].

As we previously described, the first peak of pertussis in AP Vojvodina after many years without a significant number of cases was recorded in 2012, coinciding with the introduction of enhanced pertussis surveillance and the availability of laboratory confirmation [12,14]. However, the number of cases was highest during 2023 and 2024. Notably, between 2018 and 2024, the average coverage rates for primary immunization and the first booster dose against pertussis in AP Vojvodina were 94% and 87%, respectively. Additionally, during this period, 68.4% of all cases occurred between November and March, indicating a seasonal pattern similar to that observed for other non-vaccine-preventable respiratory infections [18].

Global coverage with three doses of pertussis-containing vaccines decreased from 86% in 2019 to 83% in 2020 [26]. Despite continuous vaccine availability, fear of visiting healthcare facilities during the COVID-19 pandemic resulted in a decline in pertussis vaccine coverage in our region [12]. A decline in pertussis vaccine coverage during the four years of the COVID-19 pandemic (2020–2023) was also evident in Serbia’s neighboring countries (Bosnia and Herzegovina, Bulgaria, Croatia, Montenegro, North Macedonia, and Romania). In 2023, coverage for primary immunization with three doses of pertussis-containing vaccines (DTaP3) in these countries ranged from 73% (Bosnia and Herzegovina) to 93% (Croatia). In all these countries, the highest number of reported pertussis cases between 2000 and 2024 was recorded in either 2023 or 2024. Hungary, which also borders Serbia, reported a DTaP3 coverage of 99% in 2023; however, in 2024, 632 pertussis cases were recorded, which is several times higher than in previous years [26]. Evidence from Hungary underscores that high vaccination coverage in children does not sufficiently protect the older susceptible population. A similar resurgence of pertussis has also been reported in other European countries [23].

We found that the cumulative incidence of pertussis was highest among school-aged children aged 10–14 years. However, the accumulation in cases in this age group is primarily due to the exceptionally high number of cases recorded in the last two years compared to the entire study period. Out of all pertussis cases in patients aged 10–14 years, 60.9% (597/981) were reported between September 2023 and March 2024. During the same period, 58.4% (256/438) of cases among adolescents aged 15–19 years and 47.1% (337/716) of cases among adults aged ≥20 years were recorded. In contrast, pertussis in younger age groups (≤12 months, 1–4 years, and 5–9 years) showed a similar monthly distribution by years throughout the period from 2012 to 2024. These findings indicate that during epidemic waves of pertussis, circulation of *B. pertussis* shows a clear seasonal peak in late autumn and winter, particularly among school-aged children and adolescents, while infants are more consistently affected year-round. This pattern suggests that during pertussis outbreaks, transmission occurs more rapidly within school settings and subsequently spreads to the broader population. Consistent with our findings, a study from Catalonia covering the period from September 2023 to April 2024 reported a shift in the seasonal pattern of pertussis, with cases increasing in January and peaking in February and March. This contrasts with the period from 2011 to 2019, when most pertussis cases occurred between May and July [27]. This shift in seasonal distribution has been attributed to the impact of certain public health measures implemented during the COVID-19 pandemic, which affected the epidemiology of pertussis and other respiratory infectious diseases [27,28]. The authors of the aforementioned study also noted that in recent years, the highest incidence of pertussis was observed among children aged 10–14 years, with an incidence rate 3.4 times higher than that in infants ≤ 12 months during the 2021–2024 period. In addition to the waning vaccine-induced immunity against pertussis among school-aged children (as the last booster dose is administered at six years of age), another significant factor contributing to the discrepancy in incidence between infants and children aged 10–14 years is the introduction of maternal immunization in 2014 in Catalonia [27]. A progressive reduction in pertussis cases among infants has been observed in countries with established maternal vaccination programs [27,29,30,31]. In line with the World Health Organization recommendation issued more than ten years ago [1], by 2025, with the exception of Finland, Slovakia, Estonia, and Malta, all other European Union/European Economic Area (EU/EEA) countries will recommend pertussis immunization during pregnancy [32].

In Serbia, after being discontinued in 2001, the second booster dose was reintroduced in 2022. Additional booster doses of the pertussis vaccine for adolescents, maternal immunization against pertussis between 28 and 39 weeks of gestation during each pregnancy, as well as vaccination of HCWs in departments with a high risk of pertussis complications—such as neonatology, pediatrics, intensive care, pulmonology, obstetrics, oncology, and infectious disease units—are recommended [15,16]. However, these recommendations are still pending full implementation in practice [12].

The epidemiology of pertussis is complex and influenced by multiple factors beyond the type of vaccine used, including the pathogen’s high transmissibility, vaccination schedules and coverage, and the availability of timely laboratory diagnostics and control measures. Although countries using acellular pertussis (aP) vaccines often report higher incidence rates, direct comparisons of epidemiological data between countries are limited by differences in surveillance capacities and varying clinical and laboratory diagnostic criteria for pertussis [13,14,33,34].

Considering the specific characteristics of the studied region, our findings have several implications for public health policy. First, the resurgence of pertussis in older age groups highlights the potential benefit of introducing a booster dose during adolescence, particularly at 10–14 years of age, to reduce transmission during school years. Notably, a key finding was the temporal shift in the vaccination status of pertussis cases: While unvaccinated individuals accounted for most cases in the early surveillance period (1997–2010), the proportion of fully vaccinated cases increased substantially in later periods, especially between 2018 and 2024, when nearly half of all cases occurred in fully immunized individuals, predominantly among children aged 10–14 years. Second, the persistently high burden among infants underscores the importance of maternal vaccination during pregnancy, which has been shown to effectively reduce pertussis-related morbidity and mortality in early infancy [27,29,30,31]. Third, based on our results, in the absence of additional booster doses, outbreaks of pertussis can be expected when case numbers rise rapidly in parallel with the reopening of schools. Finally, strengthening diagnostic capacity and maintaining high-quality, age-stratified surveillance are essential for timely detection and effective control of outbreaks.

### Strengths and Limitations

While our previous publication [12] provided a broad historical overview of pertussis epidemiology in AP Vojvodina from 1948 to 2023, emphasizing long-term trends in incidence, mortality, and annual vaccination coverage, the current manuscript offers a more detailed and timely exploration of recent epidemiological patterns over the period 1997–2024. This study delves into the notable resurgence of pertussis cases observed after 2012, incorporating a thorough analysis of temporal trends and considering the potential influence of the COVID-19 pandemic on disease dynamics. By examining seasonal and monthly distributions of cases, the study sheds light on evolving transmission patterns and identifies peak periods across different age groups. A particular strength of this work lies in its focus on the shifting age-specific incidence, highlighting the increasing burden not only among infants, but also among adolescents aged 10–14 years—a trend that had not been previously addressed in detail. Furthermore, by analyzing cases according to individual vaccination status across four consecutive seven-year intervals, the study offers important insights into changes in the proportion of cases among vaccinated, partially vaccinated, and unvaccinated individuals, thereby contributing valuable information on vaccine effectiveness and potential immunity gaps within the population. Overall, this more granular and contemporary analysis provides important epidemiological evidence to inform public health strategies, including targeted immunization programs and the potential need for booster dose recommendations, as well as maternal vaccination during pregnancy. However, several limitations should be acknowledged. First, underreporting may have occurred, particularly among adolescents and adults, due to atypical or mild clinical presentations and failure to seek medical care. Second, laboratory confirmation was not consistently available in the earlier years of the study, which may have affected diagnostic accuracy. Third, although this limitation was mainly observed among individuals aged ≥20 years, a considerable proportion of cases had unknown vaccination statuses, limiting the precision of vaccine effectiveness estimates. Nonetheless, given the limited duration of vaccine-induced immunity, analyzing vaccination coverage in this age group has limited relevance for interpreting pertussis occurrence. Furthermore, we did not have data on the type (whole-cell or acellular vaccines) of pertussis vaccine administered to vaccinated individuals. Although the acellular pertussis vaccine was introduced into the national immunization program in Serbia in 2015, it is possible that some individuals born prior to that year received this vaccine via the private healthcare sector. Lastly, this analysis did not include clinical severity or outcomes such as hospitalization or complications. 

Despite these limitations, we believe that this study provides valuable evidence on the temporal, seasonal, and age-specific distribution of pertussis cases in AP Vojvodina over nearly three decades, which may help inform the optimization of pertussis prevention strategies.

## 5. Conclusions

Despite decades of high childhood vaccination coverage, pertussis has re-emerged in AP Vojvodina, with increasing incidence particularly among adolescents and infants. Our analysis of surveillance data from 1997 to 2024 reveals a shift in the age distribution of cases and a growing proportion of infections among fully vaccinated individuals, likely reflecting waning immunity following acellular pertussis vaccination. Regarding the cumulative incidence of pertussis during the 28-year study period in AP Vojvodina, our findings suggest that booster immunization in adolescence, along with routine maternal vaccination during pregnancy, could help reduce transmission, particularly to infants. The pronounced seasonal pattern, along with periodic epidemic waves, highlights the need for sustained vigilance and timely public health responses.

## Data Availability

The data that support the findings of this study are available from the corresponding authors upon reasonable request.

## Supplementary Materials

The following supporting information can be downloaded at: https://www.mdpi.com/article/10.3390/vaccines13080814/s1, Table S1: National Mandatory Immunization Program against pertussis in Serbia, 1960–2024; Figure S1: Total number of pertussis cases by months in AP Vojvodina, 1997–2024; Figure S2: Total number of pertussis cases by months and age groups (≤12 months = a; 1–4 years = b; 5–9 years = c; 10–14 years = d; 15–19 years = e, and ≥20 years = f) in AP Vojvodina, 1997–2024.
