# Supplementary material for: Resurgence of Pertussis in the Autonomous Province of Vojvodina, Serbia: Shifting Seasonality, Age Patterns, and the Need for Booster Immunization
Source: Vaccines (Basel). 2025 Jul 31;13(8):814. doi: 10.3390/vaccines13080814 (PMC12390123; doi:10.3390/vaccines13080814)
Supplement: Supplementary file 1 [file vaccines-13-00814-s001.zip › vaccines-3741263-supplementary.pdf]

Table S1: National Mandatory Immunization Program against pertussis in Serbia, 1960-2024 [10, 11, 15, 16]

Year	Activity	Vaccine type	Schedule
1960	Introduction of primo-immunization against pertussis	DTwP	2, 4, 6 months
1961	Introduction of revaccination (first booster) against pertussis	DTwP	One year after the third dose of vaccine
1981	Introduction of second booster	DTwP	At 4 years
1985	Second booster dropped	DTwP	-
1986	Second booster reintroduced	Monovalent whole-cell pertussis vaccine	At 4 years
2001	Second booster dropped	Monovalent pertussis vaccine	-
2015	DTwP vaccine officially replaced by the DTaP-IPV-Hib	DTaP-IPV-Hib	Same schedule as for DTwP vaccine
2022	Second booster reintroduced	DTaP-IPV	At 6-7 years

Legend: DTwP: whole-cell pertussis vaccine; DTaP-IPV-Hib and DTaP-IPV: acellular pertussis vaccine (this vaccine was also available on the private market in Serbia between 2001 and 2014).

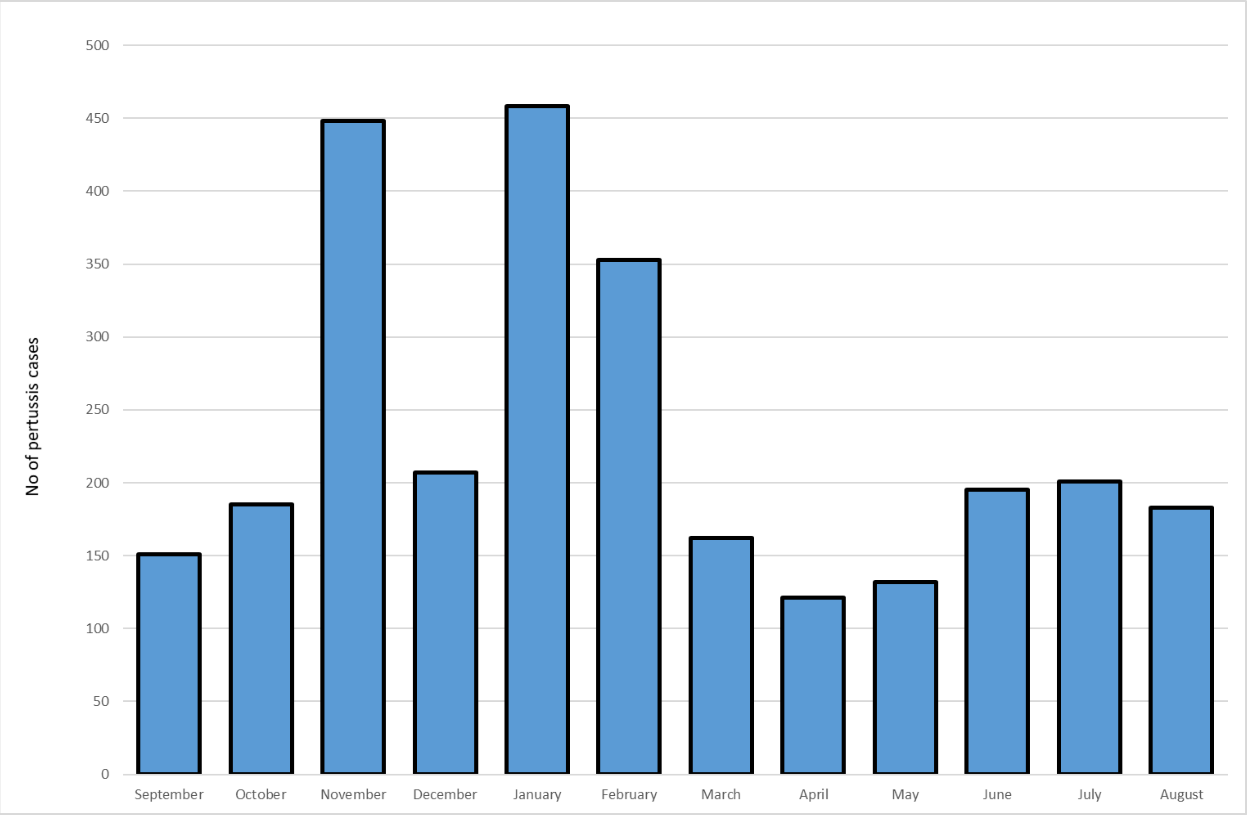

Figure S1: Total number of pertussis cases by months in Vojvodina, 1997-2024

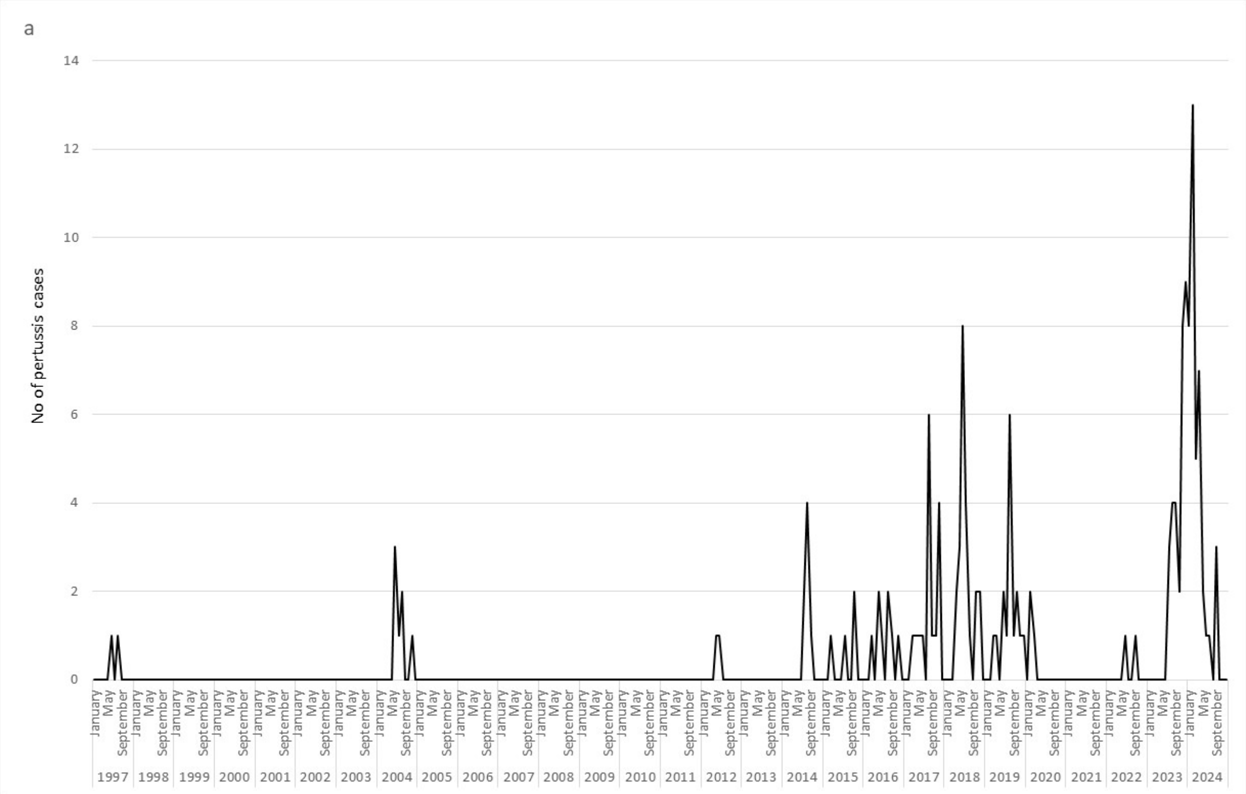
a
No of pertussis cases
1997
1998
1999
2000
2001
2002
2003
2004
2005
2006
2007
2008
2009
2010
2011
2012
2013
2014
2015
2016
2017
2018
2019
2020
2021
2022
2023
2024

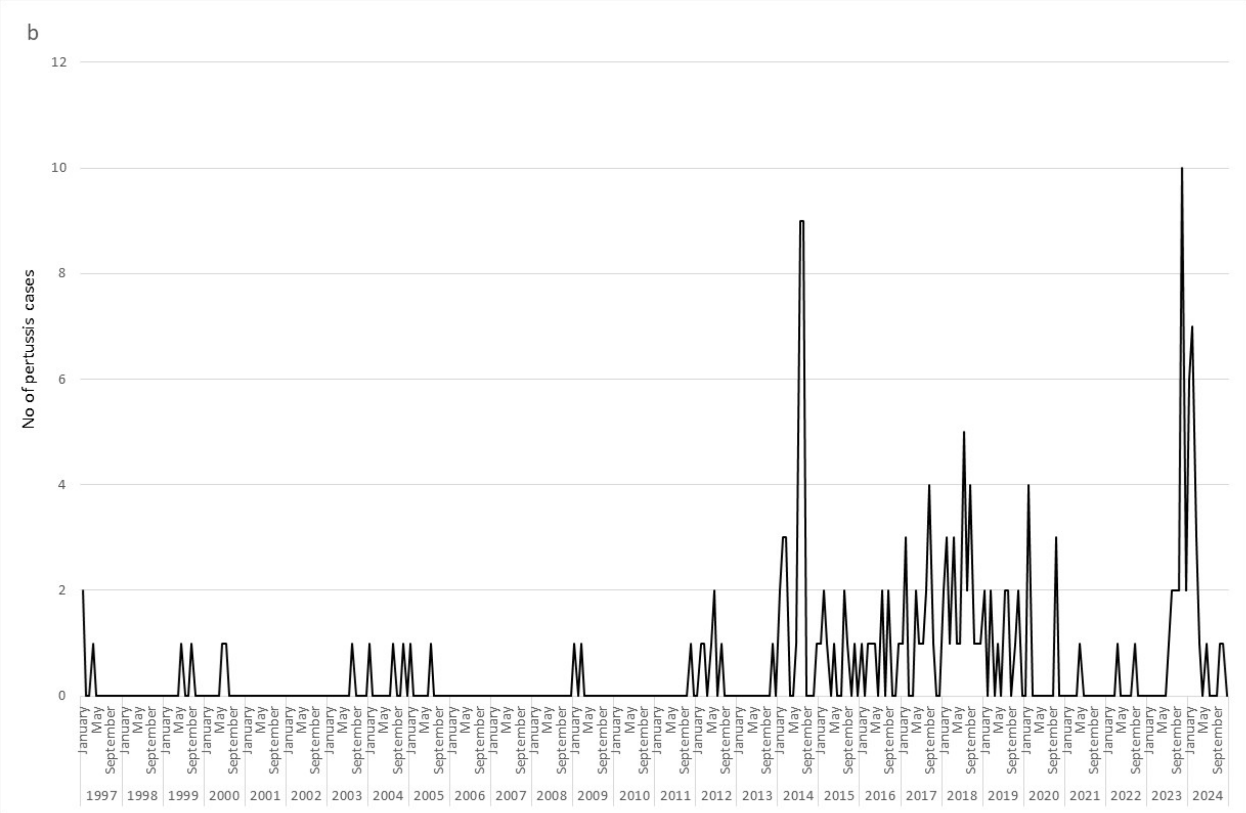
b
No of pertussis cases
1997
1998
1999
2000
2001
2002
2003
2004
2005
2006
2007
2008
2009
2010
2011
2012
2013
2014
2015
2016
2017
2018
2019
2020
2021
2022
2023
2024

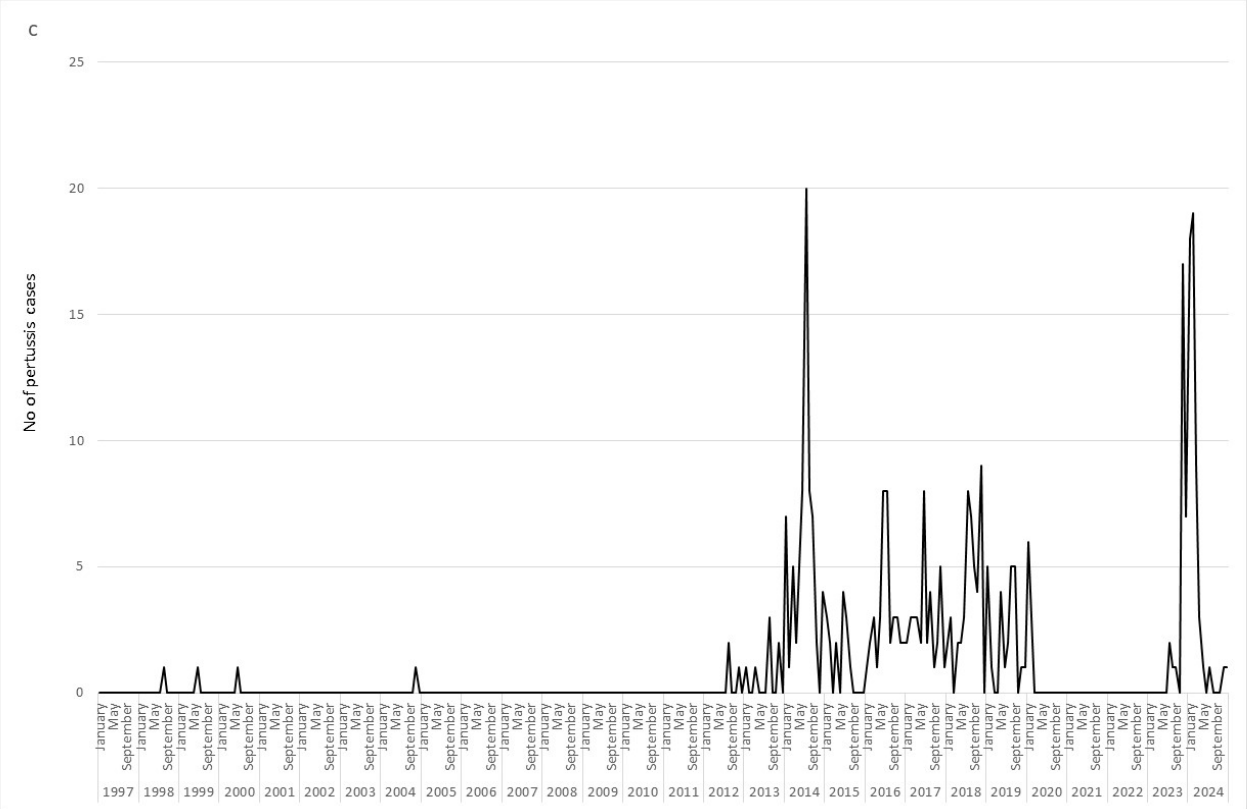
c
No of pertussis cases
0
5
10
15
20
25
January
May
September
1997 1998 1999 2000 2001 2002 2003 2004 2005 2006 2007 2008 2009 2010 2011 2012 2013 2014 2015 2016 2017 2018 2019 2020 2021 2022 2023 2024

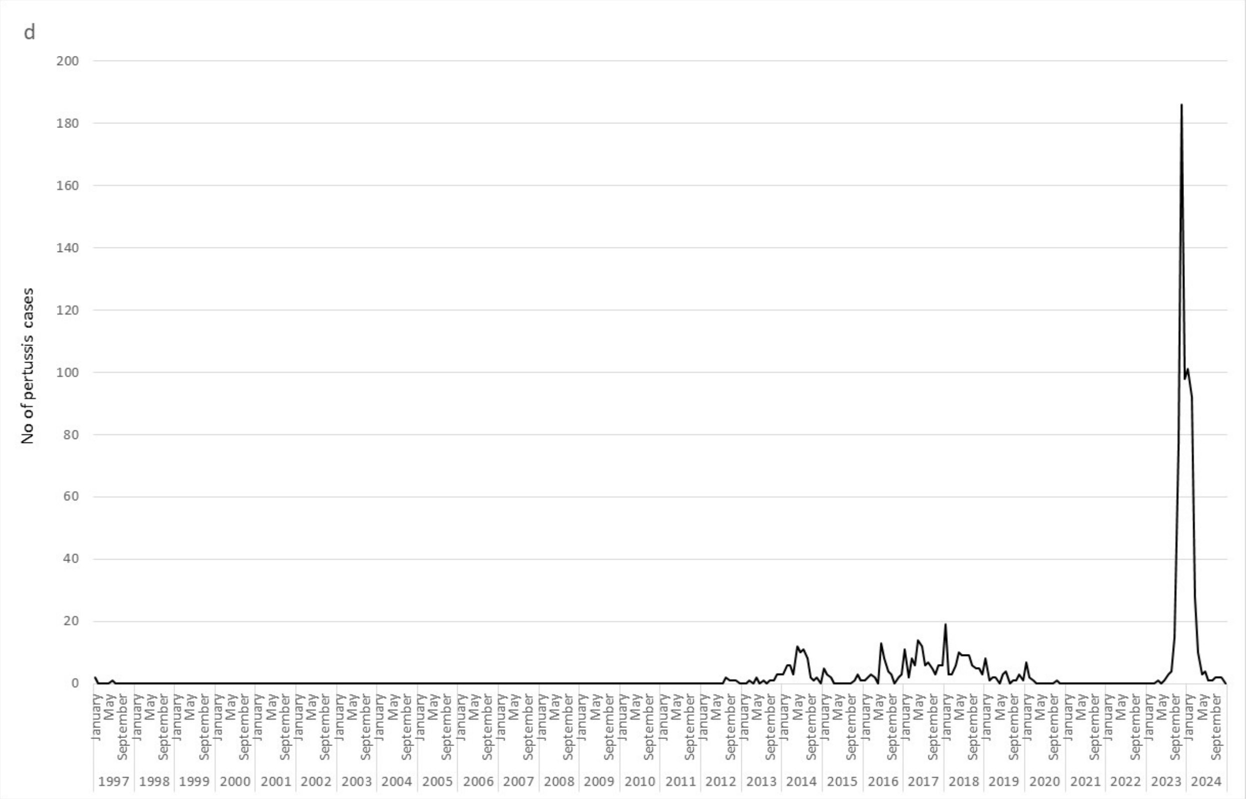
d
No of pertussis cases
0
20
40
60
80
100
120
140
160
180
200
January
May
September
1997 1998 1999 2000 2001 2002 2003 2004 2005 2006 2007 2008 2009 2010 2011 2012 2013 2014 2015 2016 2017 2018 2019 2020 2021 2022 2023 2024

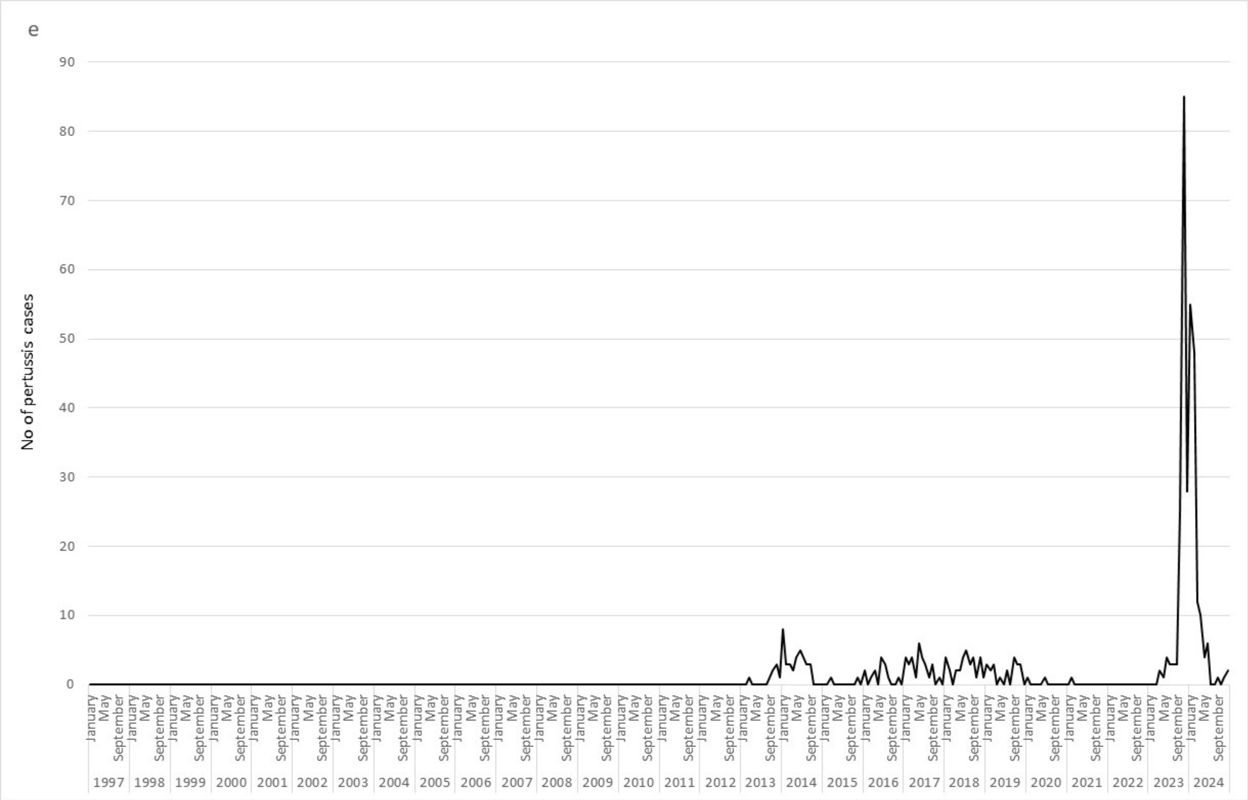

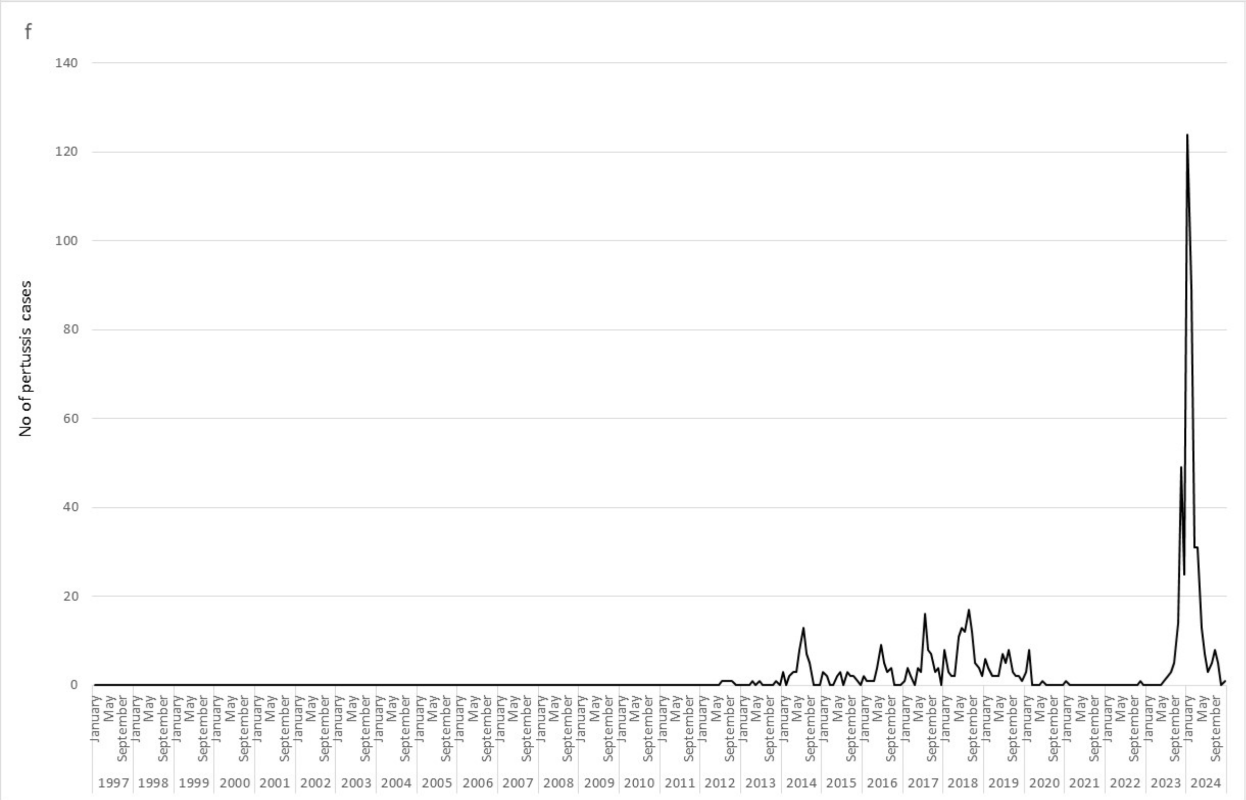

Figure S2: Total number of pertussis cases by months and age groups (≤12 months=a; 1-4 years=b; 5-9 years=c; 10-14 years=d; 15-19 years=e, and ≥20 years=f) in Vojvodina, 1997-2024
